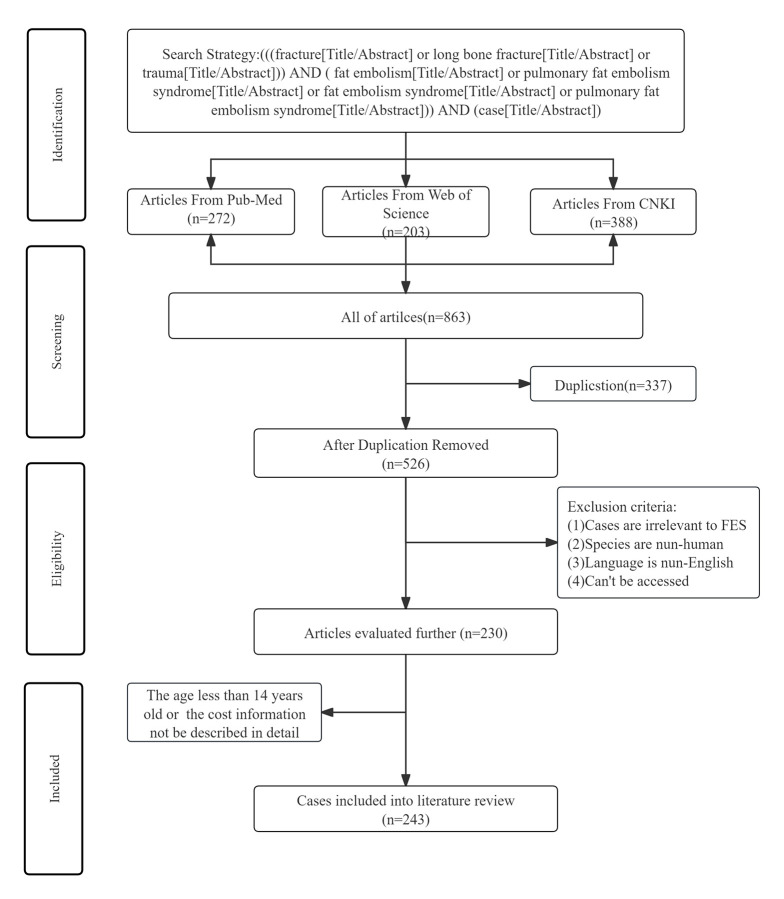# Fat embolism syndrome: Never give up – A case report and literature review

**DOI:** 10.1097/MD.0000000000043864

**Published:** 2025-08-08

**Authors:** Sisi Liu, Xiao Wu, Yun Zheng, Afang Zhang, Chengcai Dai, Linlin Pan, Qianqian Tu, Wen Dai, Wenhao Tang, Hong Zhang

**Affiliations:** a Department of Emergency Medicine, The First Affiliated Hospital of Anhui Medical University, Hefei, China.

**Keywords:** diagnosis, fat embolism syndrome, fractures, therapy, trauma

## Abstract

**Rationale:**

Fat embolism syndrome (FES) is a complex syndrome with high in-hospital mortality and the diagnosis and treatment of FES remain challenging. In this article, we present a case report as well as a comprehensive review of trauma-related cases to enhance the understanding of FES.

**Patient concerns:**

An 18-year-old young man developed a fever, tachycardia, and dyspnea about 58 hours after the trauma. Chest computed tomography (CT) scan revealed blizzard-like changes on lung window obviously different from the initial chest CT.

**Diagnoses:**

The patient’s medical history, clinical manifestations, and auxiliary examination results, especially the bronchoalveolar lavage fluid confirmed the diagnosis.

**Interventions:**

Chest CT scan demonstrated a significant absorption of the original lesions after the completion of methylprednisolone therapy for 3 days.

**Outcomes:**

The patient was successfully discharged from the hospital without any complication.

**Lessons:**

When sudden dyspnea or altered consciousness arises, the possibility of FES should be taken into account. The bronchoalveolar lavage fluid can be helpful in diagnosing and assessing the severity of pulmonary embolism. Methylprednisolone can be employed in the treatment of FES while the complications should be considered.

## 1. Introduction

Fat embolism syndrome (FES) is a rare and intricate disorder, which is manifested by respiratory distress, neurological anomalies, and a petechial rash. FES usually occurs in patients with trauma, particularly long bone fractures. Despite advancements in medicine, the diagnosis and treatment of FES remain challenging. In this context, we present a case involving a patient with long bone fractures who exhibited severe hypoxemia. The purpose of this report is to deepen the comprehension of FES.

## 2. Case presentation

**Figure 1.**
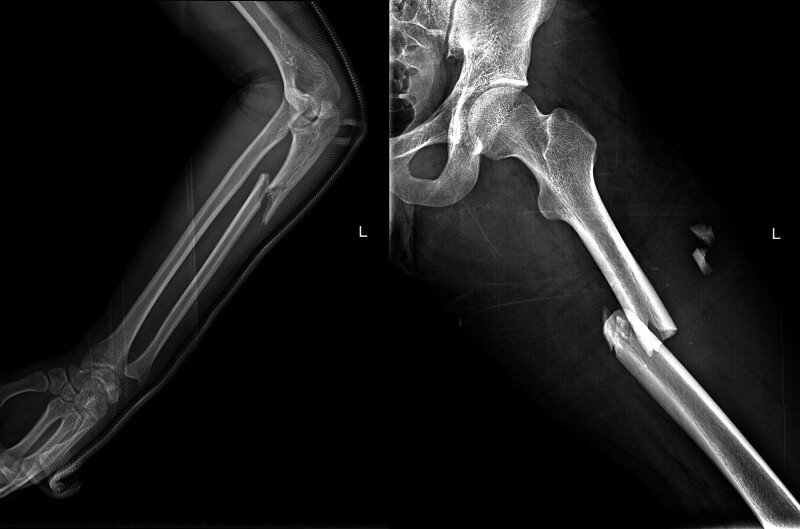
X-ray showed the fracture of the left proximal ulna (left) and the left femoral shaft (right).

**Figure 2.**
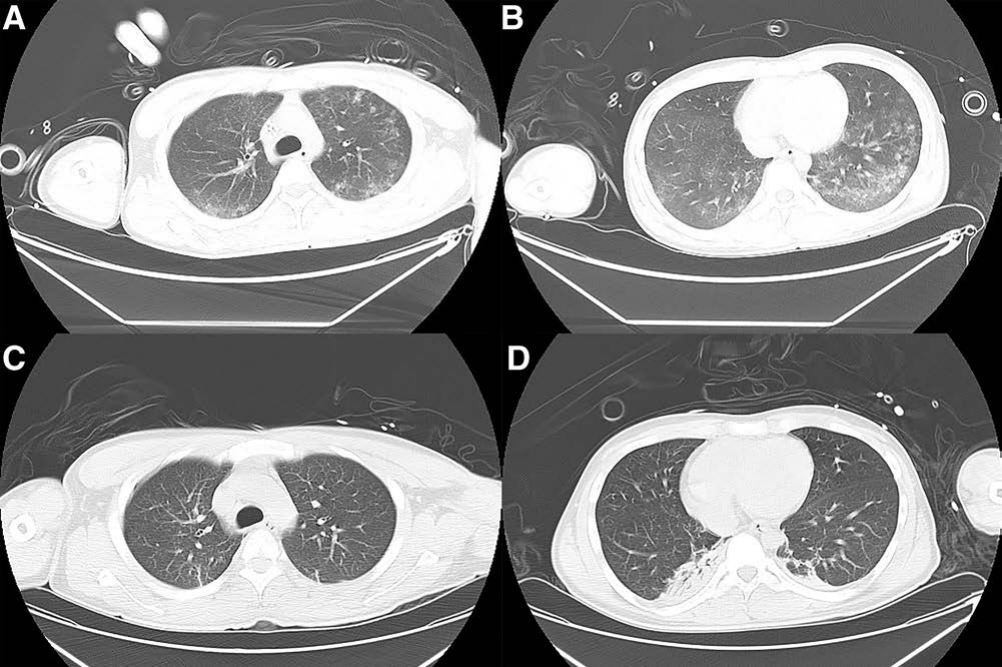
Chest CT image showed blizzard-like changes on lung window (A, B). The diagrams revealed absorption of the original lesions (C, D) after the treatment.

A young man without any basic diseases in the past 18 years was admitted to the emergency department of our hospital 2 hours after a bicycle accident. He presented with pain, swelling, and limited mobility in the left limbs. The patient had no underlying medical conditions. Upon admission, vital signs revealed a temperature of 36.8 °C, blood pressure of 121/74 mm Hg, heart rate of 105 beats/min, respiratory rate of 22 breaths/min, and blood oxygen saturation level of 99% on room air. Height of 170 cm, weight of 55 kg. The physical examination showed that the patient was conscious. His left elbow and thigh were swollen and tender, with restricted movement. X-ray examination revealed fractures of the left proximal ulna (Fig. 1, left) and the left femoral shaft (Fig. 1, right). The patient was admitted to the orthopedic department on the same day and underwent external traction on the left tibial tuberosity. About 58 hours postoperatively, the patient suddenly experienced breathing difficulties, and his peripheral blood oxygen saturation dropped to as low as 46% (in room air). He developed a fever reaching 39.1 °C, with a heart rate that peaked at 190 beats per minute. Emergency tracheal intubation was performed, and he was then transferred to the emergency intensive care unit. Arterial blood gas analysis (invasive mechanical ventilation at FiO_2_ of 40%) showed a PH of 7.377, PaCO_2_ of 42.7 mm Hg, PO_2_ of 85.9 mm Hg, HCO3^-^ of 24.2 mmol/L, with a PaO_2_/FiO_2_ ratio of 215. Computed tomographic pulmonary angiography showed no signs of pulmonary embolism, while the main pulmonary artery was slightly widened with 29.8 mm in diameter. Chest computed tomography (CT) scan revealed blizzard-like changes on lung window (Fig. 2A and B). A CT scan of the head revealed no evident abnormal findings. Limbs vascular ultrasound was unremarkable. Echocardiography indicated a decreased compliance in the left ventricle, and the left ventricular ejection fraction was 60%.

**Figure 3.**
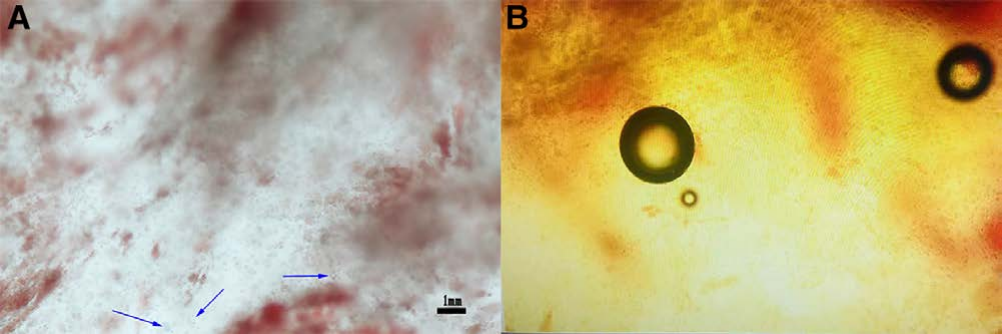
Lipid droplets under the microscope (left 100×, right 400×).

Initially, pulmonary embolism was considered the most likely diagnosis due to the patient’s medical history, clinical manifestations, and auxiliary examination results. Anticoagulant therapy was initiated first. Meanwhile, human albumin was administered for 4 consecutive days, and low molecular weight dextran was given for 3 days. After a comprehensive multidisciplinary discussion, a microscopic examination of the bronchoalveolar lavage fluid (BALF) was conducted. The results showed a significant number of lipid droplets (Fig. 3), which led to a conclusive diagnosis of pulmonary fat embolism syndrome (FES). Promptly initiated, with a dosage regimen of 500 mg per day for the initial 3 days, subsequently reduced to 300 mg per day and then to 100 mg per day for a cumulative treatment duration of 5 days. Three days subsequent to the completion of glucocorticoid therapy, a follow-up chest CT scan demonstrated a significant absorption of the original lesions (Fig. 2C and D). On the 18th day after the trauma, the invasive ventilator was withdrawn, and the patient was transferred to general ward the next day. Approximately 1 month later, the patient was successfully discharged from the hospital.

## 3. Discussion

FES, which is marked by respiratory distress, neurological irregularities, and petechial rash, mainly happens in trauma patients who have suffered long bone fractures. We performed a literature review by searching the published case report of fat embolism following fractures or trauma from the PubMed (1960–November 2024), Web of Science (1960–November 2024), and CNKI (1960–November 2024) in English using the following search strategy: “(((fracture[Title/Abstract] or long bone fracture[Title/Abstract] or trauma[Title/Abstract])) AND (fat embolism[Title/Abstract] or pulmonary fat embolism syndrome[Title/Abstract] or fat embolism syndrome[Title/Abstract] or pulmonary fat embolism syndrome[Title/Abstract])) AND (case[Title/Abstract]).” The age less than 14 years old or non-human cases were excluded, resulting in the final inclusion of 243 patients. The detailed procedure of literature screening is presented in Figure S1, Supplemental Digital Content, https://links.lww.com/MD/P660. The demographic and clinical characteristics of all patients were summarized in Table 1. Recent research has indicated that the incidence of FES in patients undergoing surgery after traumatic injuries is 0.07%, with an in-hospital mortality rate of 13.8%, which is significantly higher compared to patients without fat embolism. Additionally, the length of hospital stay and associated costs were also found to be increased.^[1]^ Consistent with these findings, our review also demonstrated an in-hospital mortality rate of 19.3% for patients with FES. The absence of typical clinical syndrome, the need for specialized laboratory examinations, the challenges in differential diagnosis, and the high mortality rate make FES a significant issue, imposing substantial economic and public health burdens.

**Table 1:** Clinical characteristics and outcome of 243 patients with fat embolism syndrome.

Characteristics	Only long bone fracture (n = 140)	Non-long bone fracture (n = 27)	Mixed fracture (n = 67)	Trauma without fracture (n = 9)
Sex, n (%)				
Male	103/140 (73.6)	12/27 (44.4)	49/67 (73.1)	8/9 (88.9)
Female	37/140 (26.4)	15/27 (55.6)	18/67 (26.9)	1/9 (11.1)
Age, years, median (range)	26 (15.0–93.0)	66 (14.0–89.0)	27 (14.0–93.0)	23 (14.0–88.0)
Clinical manifestations, n (%)				
Dyspnea or hypoxemia	86/105 (81.9)	14/15 (93.3)	43/51 (84.3)	6/6 (100.0)
Neurologic abnormalities	93/105 (88.6)	15/16 (93.8)	46/49 (93.9)	5/6 (83.3)
Petechial rash	42/69 (60.9)	6/10 (60.0)	21/35 (60.0)	0
Fever	36/46 (78.3)	3/6 (50.0)	16/20 (80.0)	3/3 (100.0)
Tachycardia	52/58 (89.7)	8/8 (100.0)	24/27 (88.9)	6/6 (100.0)
Retinal changes	13/21 (61.9)	1/1 (100.0)	0	0
Jaundice	1/4 (25.0)	0	1/2 (50.0)	0
Laboratory examination, n (%)				
Renal changes	10/20 (50.0)	1/1 (100.0)	3/10 (30.0)	0
Anemia	34/38 (89.5)	4/5 (80.0)	23/26 (88.5)	1/1 (100.0)
Thrombocytopenia	33/38 (86.8)	5/5 (100.0)	21/26 (80.8)	0
High ESR	4/4 (100.0)	0	2/2 (100.0)	0
Fat macroglobulinemia	0	1/1 (100.0)	3/3 (100.0)	0
Organ of embolism, n (%)				
Lung	75/140 (53.6)	13/27 (48.1)	36/67 (53.7)	7/9 (77.8)
Brain	80/140 (57.1)	17/27 (63.0)	36/67 (53.7)	3/9 (33.3)
Kidney	4/140 (2.9)	0	1/67 (1.5)	2/9 (22.2)
Eyes	8/140 (5.7)	0	1/67 (1.5)	0
Liver	0	0	0	1/9 (11.1)
Pancreas	0	0	0	1/9 (11.1)
Heart	0	0	1/67 (1.5)	0
Embolism of lower extremity vein	4/140 (2.9)	0	4/67 (6.0)	0
Embolism of subclavian vein	1/140 (0.7)	0	0	0
Embolism of inferior vena cava	1/140 (0.7)	0	0	0
Uncertain	0	0	1/67 (1.5)	0
Image findings for diagnosis, n (%)				
Chest X-ray	48/65 (73.9)	4/5 (80.0)	20/27 (74.1)	3/3 (100.0)
Chest CT	40/52 (76.9)	1/3 (33.3)	6/13 (46.2)	0
Brain MRI	59/61 (96.7)	8/8 (100.0)	29/31 (93.6)	0
Ultrasound cardiogram				
Right ventricular abnormality	4/62 (6.5)	1/9 (11.1)	11/34 (32.4)	2/4 (50.0)
Pulmonary hypertension	2/62 (3.2)	1/9 (11.1)	2/34 (5.9)	0
Patent foramen ovale	3/62 (4.8)	2/9 (22.2)	4/34 (11.8)	0
CTPA	11/40 (27.5)	2/6 (33.3)	2/14 (14.3)	0
Treatment, n (%)				
Corticosteroid	31/35 (88.6)	1/1 (100.0)	2/2 (100.0)	2/2 (100.0)
Albumin	6/6 (100.0)	0	1/1 (100.0)	0
Anticoagulant therapy	28/30 (93.3)	3/3 (100.0)	12/13 (92.3)	0
Invasive mechanical ventilation	87/118 (73.3)	13/17 (76.5)	55/62 (88.7)	4/5 (80.0)
ECMO	0	0	8/8 (100.0)	0
Prognosis, n (%)				
Survivor	119/138 (86.2)	15/26 (57.7)	50/65 (76.9)	8/9 (88.9)
Non-survivor	19/138 (13.8)	11/26 (42.3)	15/65 (23.1)	1/9 (11.1)
Prognosis do not be mentioned	2/140 (1.4)	1/27 (3.7)	2/67 (3.0)	0

CT = computed tomography, CTPA = computed tomographic pulmonary angiography, ECMO = extracorporeal membrane oxygenation, ESR = erythrocyte sedimentation rate, FES = fat embolism syndrome, MRI = magnetic resonance imaging.

Fat embolism was initially reported over a century and a half ago.^[2]^ FES represents a rare and specific clinical condition that must not be mistaken for simple fat embolism. The entry of fat globules into the bloodstream is believed to stem from a complex interplay between mechanical and biochemical elements. Subsequently, these globules migrate to crucial organs like the brain and lungs. These fat globules trigger microcirculatory disruptions, thus leading to a systemic pathophysiological reaction. Pulmonary fat embolism emerges as the predominant clinical manifestation. FES usually occurs in 12 to 72 hours post-injury, especially follows long bone fractures. In this literature review, the onset time of FES was detailed for approximately 136 cases. The majority of patients exhibited FES symptoms within 24 hours of their initial admission, with 50 patients experiencing particularly severe discomfort within 12 hours, most of whom (78%) had long bone fractures. Additionally, 17 patients showed no FES symptoms prior to 72 hours.

The diagnostic process for FES remains notably challenging. A combination of the Gurd and WilSon^[3]^ and the Lindeque criteria^[4]^ is the most widely accepted diagnostic approach for FES^[5]^ (Table 2). In our extensive review of previous research, it became apparent that patient clinical manifestations, notably dyspnea, neurologic anomalies, and tachycardia, in conjunction with laboratory indicators such as anemia and thrombocytopenia, are crucial elements in diagnosing FES. However, 26 cases exhibited no clinical symptoms or abnormalities in routine blood tests and could only be diagnosed through imaging examinations or final autopsies. Among these patients, 11 had preexisting comorbidities, such as hypertension, coronary artery disease, and chronic lung disease. Notably, more than half of the patients were over 60 years old. These findings highlight the ongoing challenges in the clinical diagnosis of FES.

**Table 2:** Diagnosis criteria for fat embolism syndrome.

		Points
Gurd and Wilson*		
Major criteria		
	Respiratory insufficiency	–
	Cerebral involvement	–
	Petechial rash	–
Minor criteria		
	Fever (>38.5 °C)	–
	Tachycardia (>110 beats/min)	–
	Retinal changes	–
	Jaundice	–
	Renal changes	–
	Anemia	–
	Thrombocytopenia	–
	High erythrocyte sedimentation rate (>71 mm/h)	–
	Fat macroglobulinemia	–
Schonfeld et al†		
	Petechia	5
	Chest radiograph changes	4
	Hypoxemia	3
	Fever (>38.0 °C)	1
	Tachycardia (>120 beats/min)	1
	Tachypnea (>30 beats/min)	1
	Confusion	1
Lindeque et al‡		
	Sustained PaO_2_ (<8 kPa)	–
	Sustained PaCO_2_ (>7.3 kPa) or PH <7.3	–
	Sustained respiratory rate (>35/min)	–
	Increased work of breathing, dyspnea, tachycardia, anxiety	–

* Two major criteria or 1 major criterion plus at least 4 minor criteria.

† More than 5 scores.

‡ At least one of criteria for diagnosis of FES.

In this case report, the patient experienced respiratory distress around 58 hours after sustaining left long bone fractures. The patient had an elevated heart rate reaching up to 190 beats per minute and was also accompanied by a fever of 39.1 °C. Chest CT clearly revealed typical pulmonary embolism changes, yet head CT indicated no signs of intracranial hemorrhage or other lesions. Blood examination demonstrated no significant reduction in hemoglobin or platelet levels. Moreover, there were no abnormalities regarding vision or urine. Despite the absence of a confirmed diagnosis of FES for this patient, the preliminary diagnosis considered the possibility of pulmonary embolism based on the patient’s history and clinical features. However, the nature of the embolic material, whether thrombus or fat, remained uncertain. Consequently, ultrasound examinations of the limbs and heart were conducted, which did not detect any thrombi. The pulmonary fat embolism was clarified finally owing to the fat droplets found in the BALF.

Pulmonary and cerebral embolisms are frequently observed in clinical practice. Ocular fat embolism can be diagnosed through fundoscopy. Additionally, a few case reports have documented fat embolism involvement of the kidney, liver, pancreas, and heart through autopsy. In our comprehensive literature review, we identified 131 cases of pulmonary fat embolism and 136 cases of cerebral fat embolism. Among these cases, 75 underwent cardiac ultrasound examinations, with 11 demonstrating evidence of anatomical shunt. Upon entering the blood circulation, lipid particles initially reach the right heart. Typically, they must transit through the pulmonary circulation. However, in a small subset of patients, these particles may bypass this pathway due to the presence of structural anomalies such as patent foramen ovale, atrial septal defect, ventricular septal defect, or intrapulmonary shunt, thereby directly accessing the cerebral arteries and inducing cerebral fat embolism. Out of the 136 cases reviewed, 38 patients were found to have both cerebral and pulmonary fat embolism. Notably, 68 cases presented with symptoms of dyspnea or hypoxemia. Given these findings, we speculate that a few cases of pulmonary embolism may have been overlooked due to the lack of specific diagnostic methods. This underdiagnosis could have contributed to the discrepancy in the number of identified cases between pulmonary and cerebral fat embolism. The detection of small fat droplets in BALF is critical for the diagnosis of pulmonary fat embolism. The presence of fat droplets in BALF suggests that microfat particles have infiltrated the pulmonary vasculature and formed emboli. Consequently, we believe that invasive procedures such as bronchoalveolar lavage can provide valuable diagnostic insights in cases where clinical diagnosis is challenging.

There are still some controversies over the prevention of FES. Some studies suggest that early open reduction and internal fixation following trauma can reduce the incidence of FES, while other research have found no significant relationship between the timing of surgery and the occurrence of FES.^[6,7]^ In this literature review, a total of 241 patients were included in the description of surgical procedures, with 63% exhibiting fat embolism prior to the final fracture fixation (152 cases vs 89 cases). There was no significant difference in prognosis between patients with FES who underwent surgery and those who did not. However, as all the patients selected for this study had already developed fat embolism, it was not possible to explore the relationship between early surgical intervention and the prevention of FES.

When fat droplets enter the bloodstream from the bone marrow, they can obstruct blood vessels and disrupt local blood supply, resulting in a ventilation–perfusion mismatch in the lungs. Additionally, these fat particles can stimulate endothelial cells to release lipase, thereby increasing the levels of free fatty acids in circulation and causing vascular endothelial damage. The resultant endothelial injury further triggers a cascade of inflammatory responses, ultimately leading to acute respiratory distress syndrome. Human serum albumin, dextran and corticosteroids may be effective in the treatment of FES. Albumin can bind to free fatty acids, while corticosteroids can stabilize lysosomes and capillary membranes, thereby reducing blood capillary permeability. A meta-analysis have shown the benefits of corticosteroids in FES treatment.^[8]^ In this case, we administered human serum albumin, dextran and high-dose methylprednisolone without the complications of gastrointestinal hemorrhage or severe infection. subsequent chest CT scan showed significant resolution of lung lesions. The literature review also revealed that the majority of patients diagnosed with FES received treatments such as corticosteroids, albumin administration, anticoagulation therapy, and invasive mechanical ventilation.

Respiratory support therapy is crucial for patients experiencing dyspnea. In this case, we utilized invasive mechanical ventilation, which helped maintain the patient’s oxygenation index at an acceptable level. Ultimately, after intensive treatment, the patient was successfully extubated, transferred back to the regular ward, and discharged from the hospital approximately 1 month later. In patients with severe FES, extracorporeal membrane oxygenation treatment may be considered when the mechanical ventilation and conventional pharmacotherapy prove ineffective, as it has been shown to more effectively improve cardiac and pulmonary function.^[9]^ Upon reviewing the literature, extracorporeal membrane oxygenation treatment was employed in only 8 cases involving mixed fractures. Unfortunately, 2 of these patients succumbed to disseminated intravascular coagulation and ischemic encephalopathy following cardiac arrest.

## 4. Conclusions

When sudden dyspnea or altered consciousness arises, the possibility of FES should be taken into account. The detection of lipid droplets in the BALF can be beneficial for clinical diagnosis. BALF analysis combined with pulmonary imaging examinations may be helpful in diagnosing and assessing the severity of pulmonary embolism due to their high specificity and sensitivity. Methylprednisolone can be employed in the treatment of FES. However, it is of utmost importance to gradually reduce the dosage while closely monitoring for any potential complications.

## Acknowledgments

The authors thank all the subjects who participated in this study.

## Author contributions

**Conceptualization:** Yun Zheng, Hong Zhang.

**Data curation:** Xiao Wu, Yun Zheng, Afang Zhang, Chengcai Dai, Linlin Pan, Qianqian Tu, Wen Dai, Wenhao Tang.

**Funding acquisition:** Hong Zhang.

**Writing – original draft:** Sisi Liu.

**Writing – review & editing:** Sisi Liu, Hong Zhang.

## Supplementary Material

**Figure:**